# The Role of Tumor Microenvironment in Multiple Myeloma Development and Progression

**DOI:** 10.3390/cancers13020217

**Published:** 2021-01-09

**Authors:** Almudena García-Ortiz, Yaiza Rodríguez-García, Jessica Encinas, Elena Maroto-Martín, Eva Castellano, Joaquín Teixidó, Joaquín Martínez-López

**Affiliations:** 1 Hematology Department, Hospital 12 de Octubre, i+12, CNIO, CIBERONC, ES 28041 Madrid, Spain; a.garcia.imas12@h12o.es (A.G.-O.); jessienc@ucm.es (J.E.); elemarot@ucm.es (E.M.-M.); ecaste03@ucm.es (E.C.); 2 Department of Molecular Biomedicine, Centro de Investigaciones Biológicas, CSIC, ES 28040 Madrid, Spain; yaiza.rodriguez@cib.csic.es (Y.R.-G.); joaquint@cib.csic.es (J.T.); 3 Medicine Department, Medicine School, Universidad Complutense de Madrid, ES 28040 Madrid, Spain

**Keywords:** multiple myeloma, tumor microenvironment, immunosuppression, adhesion molecules, migration, bone marrow niche

## Abstract

**Simple Summary:**

Multiple Myeloma (MM) is a hematologic malignancy caused by aberrant plasma cell proliferation in the bone marrow (BM) and constitutes the second most common hematological disease after non-Hodgkin lymphoma. The disease progression is drastically regulated by the immunosuppressive tumor microenvironment (TME) generated by soluble factors and different cells that naturally reside in the BM. This microenvironment does not remain unchanged and alterations favor cancer dissemination. Despite therapeutic advances over the past 15 years, MM remains incurable and therefore understanding the elements that control the TME in MM would allow better-targeted therapies to cure this disease. In this review, we discuss the main events and changes that occur in the BM milieu during MM development.

**Abstract:**

Multiple myeloma (MM) is a hematologic cancer characterized by clonal proliferation of plasma cells in the bone marrow (BM). The progression, from the early stages of the disease as monoclonal gammopathy of undetermined significance (MGUS) and smoldering multiple myeloma (SMM) to MM and occasionally extramedullary disease, is drastically affected by the tumor microenvironment (TME). Soluble factors and direct cell–cell interactions regulate MM plasma cell trafficking and homing to the BM niche. Mesenchymal stromal cells, osteoclasts, osteoblasts, myeloid and lymphoid cells present in the BM create a unique milieu that favors MM plasma cell immune evasion and promotes disease progression. Moreover, TME is implicated in malignant cell protection against anti-tumor therapy. This review describes the main cellular and non-cellular components located in the BM, which condition the immunosuppressive environment and lead the MM establishment and progression.

## 1. Introduction

Multiple Myeloma (MM) is a hematologic malignancy characterized by clonal proliferation of plasma cells in the bone marrow (BM) and constitutes the second most common hematological disease after non-Hodgkin lymphoma [1]. This illness normally evolves from premalignant states called monoclonal gammopathy of undetermined significance (MGUS), with or without intermediate stages as smoldering multiple myeloma (SMM) or solitary plasmacytoma, and eventually progresses to plasma cell leukemia or extramedullary myeloma [2]. The transition among the stages of the disease and its resistance to treatment are regulated by the BM microenvironment. Cellular and non-cellular components of MM BM niche contribute to the generation of an immunosuppressive tumor microenvironment (TME), which supports MM cell growth and survival. In this review, we update the mechanisms of myeloma cell trafficking and the involvement of BM mesenchymal stromal cells and the immune compartment in the establishment and exacerbation of the disease.

## 2. Myeloma Cell Trafficking

**Figure 1:**
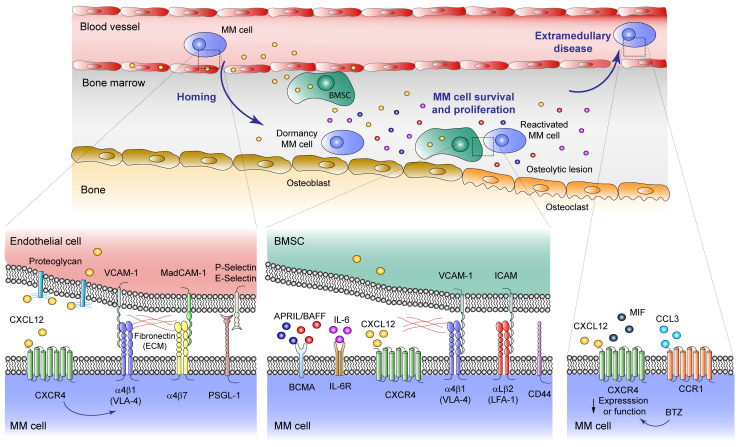
Malignant plasma cell trafficking events in multiple myeloma (MM) development. MM plasma cells reach the bone marrow (BM) niche through sinusoids, proliferate favored by the tumor microenvironment (TME) and, occasionally, egress to the circulation causing extramedullary disease. Homing: The interaction of the CXCL12 chemokine with its CXCR4 receptor, mediates homing, lodging and retention of both normal and malignant plasma cells into the BM. CXCL12–CXCR4 interaction upregulates the activity of the α4β1 integrin, allowing high binding to its ligand VCAM-1 expressed on the BM microvasculature. Other important adhesion molecules mediating MM cell homing into the BM are the α4β7 integrin, a receptor for MAdCAM-1 and fibronectin, and PSGL-1 which interacts with P- and E-selectin expressed on the endothelial cell surface. MM cell survival and proliferation: Inside the BM milieu, BM mesenchymal stromal cells (BMSC) secrete high levels of CXCL12 that, along with α4β1, α4β7, and αLβ2 integrins, as well as CD44, are important for anchoring and retention of MM cells into BM niches. During this stage, survival and proliferation of malignant plasma cells is contributed by two main soluble mediators, a proliferation-inducing ligand (APRIL) and B-cell activating factor (BAFF), which bind B-cell maturation antigen (BCMA) in the cancer cell surface, and IL-6, whose receptor is also expressed on MM cells. In MM patients there is a pathological osteolineage imbalance with reduced osteoblasts in favor of osteoclasts which produce lytic lesions. BM osteoblastic niche facilitates the dormancy of MM cells, while osteoclasts induce MM cell reactivation. Extramedullary disease: In this context, cancer cells become independent from the TME, and CXCR4 function or expression is downregulated, an event that also occurs after bortezomib (BTZ) treatment. Macrophage migration inhibitory factor (MIF) can also bind to CXCR4, inducing the expression of adhesion molecules. Expression of CCR1 chemokine receptor is linked to MM plasma cell circulation.

Different trafficking events of MM cells allow them to reach and colonize distinct BM niches, eventually recirculate, and finally egress from the BM during the extramedullary stages of the disease (Figure 1) [3,4]. Although it has not been completely elucidated, it is likely that normal plasma cells and MM cells use the BM sinusoids as an entry route into the BM, similarly to the hematopoietic stem cells (HSC) entry gates [5,6,7,8]. A main molecule mediating homing, lodging, and retention of both normal and malignant plasma cells into the BM is the chemokine receptor CXCR4, which interacts with CXCL12, a chemokine highly expressed in the BM microenvironment [3,4,5,8,9,10,11,12]. Notably, blocking CXCL12–CXCR4 interaction with the CXCR4 inhibitor plerixafor (AMD3100) disrupts MM cell contacts with the BM microenvironment [13], leading to MM cell mobilization into the circulation [14]. Among other signaling responses, the interaction of CXCL12 with CXCR4 on the surface of MM cells upregulates the activity of the α4β1 integrin, allowing high binding to its ligand VCAM-1 expressed on the BM microvasculature [15,16,17]. This adhesive event represents a key step in MM cell trafficking into the BM milieu and likely plays key roles during MM cell recirculation.

Two other important adhesion molecules mediating MM cell homing into the BM are the α4β7 integrin, a receptor for MAdCAM-1 and fibronectin, and CD44 [18,19,20]. In addition, MM cell attachment to the BM microvasculature is also contributed by P- and E-selectin and their ligands. Thus, the P-selectin glycoprotein ligand-1 (PSGL-1) is expressed on the surface of MM cells [21,22], playing key roles in the very initial interactions of MM cells with BM endothelial cells [22], by facilitating malignant plasma cell rolling on P-selectin expressed by the microvasculature [16]. The involvement of E-selectin during MM cell homing to the BM has been demonstrated using inhibitors of sialyltransferase ST3Gal-6, an enzyme required for the generation of E-selectin ligands [23,24], and by E-selectin-blocking antibodies [16].

Once inside the BM milieu, the α4β1 integrin is also important for anchoring the MM cells to BM niches, and for retaining them in the BM TME [16,25,26,27,28]. α4β1-dependent MM cell attachment was early shown to contribute to MM progression in in vivo models [29,30]. Its interaction with both VCAM-1 and fibronectin present in the BM triggers MM cell signaling leading to stimulation of their growth involving interleukin-6 (IL-6) [25,31,32], and promoting cell adhesion-mediated drug resistance [33,34]. At this stage, MM cell attachment to the BM microenvironment is also facilitated by α4β7- and by CD44-dependent adhesion, overall contributing to the retention of MM cells in the BM [18,19,20,25,35].

The final phases in MM involve egress of myeloma cells from the BM to the bloodstream to colonize different organs, once they become independent from growth signals provided by the BM milieu, a condition named extramedullary disease [4]. Decreased CXCR4 expression and function represents a possible candidate event inducing a reduction in MM cell retention associated with egress from the BM. In line with this model, treatment of MM cells with the proteasome inhibitor bortezomib decreases CXCR4 expression, which might contribute to MM cell migration from the BM and promote extramedullary disease [36], an unwanted clinical response. Furthermore, macrophage migration inhibitory factor (MIF) is also capable of binding CXCR4 [37], and its silencing causes downregulation of MM cell adhesion to BM stroma and leads to extramedullary disease [38]. The chemokine receptor CCR1 is also expressed on MM cells [12,39], and its expression is associated with increased circulating MM cells [40]. Interestingly, its ligand CCL3 inhibited MM cell migration towards CXCL12 [40], raising the possibility that the CCL3–CCR1 axis might actively promote MM cell exit from the BM, possibly by competition with retention signals from the CXCR4–α4β1 interaction.

## 3. Bone Marrow Niche

### 3.1. Bone Marrow Niche and Stromal Cells

The BM harbors hematopoietic and non-hematopoietic cells, as well as non-cellular components such as extracellular matrix (ECM) proteins and soluble factors, which work together to maintain the HSC pool and its descendants, the lineage-committed hematopoietic progenitor cells (HPCs) [41,42,43]. The non-hematopoietic cell pool includes mesenchymal stem cells (MSC) and their descendant stromal cells, as well as endothelial cells and pericytes, adipocytes, osteolineage cells and osteoclasts, and sympathetic neurons with their associated Schwann cells. Two HSC niches supporting normal hematopoiesis, the vascular and the endosteal niches, contain different cellular types, express distinct soluble factors required for HSC maintenance, retention, and proliferation, and also include HPCs which can also regulate the HSC compartment [41,42,43]. Yet, as the endosteal region of the BM is highly vascularized, it is likely that some cell type overlap exists between the endosteal and vascular HSC niches. A large portion of HSC resides perivascularly mainly surrounding fenestrated sinusoidal blood vessels, which represent important sites of migration between blood and BM [42,44]. Several perivascularly-located BM mesenchymal stromal cell types have been identified, including CXCL12-abundant reticular (CAR) cells, leptin-receptor (LPR^+^) expressing cells, *Nes*-Cre^ER^, and *NG2*-Cre^ER^ cells [45]. These cells provide key factors for HSC retention, maintenance and proliferation in defined niches, such as CXCL12 and SCF (also named c-kit ligand), both expressed by LPR^+^ perivascular stroma and by endothelial cells [41,42,45]. Furthermore, these mesenchymal stromal cells express adhesive molecular anchors for HSC, including α4β1 [46,47,48] and α5β1 integrin ligands [48,49,50], CD44 [48,51] and E-selectin [52,53]. Other soluble factors modulating but likely not required for HSC maintenance are bone morphogenetic proteins (BMP), IL-6, fibroblast growth factor (FGF) and Notch ligands [54,55,56,57,58,59], whereas transforming growth factor-β (TGF-β) induces HSC quiescence and self-renewal [60].

Other cell populations influencing the HSC niche include HSC descendants such as osteolineage cells, macrophages and megakaryocytes [41,42,45]. Moreover, BM skeletal stem cells (SSC) capable of generating colony-forming units-fibroblast (CFU-Fs) secrete HSC niche factors including angiopoietin 1, and Nestin^+^ BM stromal cells or those expressing SCF or CXCL12 are enriched for CFU-Fs and regulate the formation of HSC niches [61,62,63,64]. Macrophages control CXCL12 expression in the BM environment and induce HSC mobilization [65,66], whereas megakaryocytes have been shown to associate with HSC at sinusoids [67,68,69]. Whether HSC and their progeny occupy identical, distinct, or overlapping spatial locations in the BM is actively being investigated. As macrophages and megakaryocytes represent HSC niche constituents, it could be proposed that HSC and their descendants take up overlapping spaces and share key soluble factors for differentiation [43].

Two other elements controlling HSC function are the oxygen tension and the sympathetic nerves [41,42,43]. The HSC niche is highly hypoxic, causing stabilization of the hypoxia-inducible factor 1-α (HIF-1α), which transcriptionally activates the expression of CXCL12 and CXCR4, therefore indirectly contributing to HSC retention [70,71,72,73]. Besides being vascularized, the BM is highly innervated, especially next to arterioles [74]. Nerve fibers and non-myelinating associated Schwann cells are required for regeneration of hematopoiesis after chemotherapy [60,75], and are needed for regulation of the circadian clock that controls the daily mobilization of HSC into blood, likely by modulating CXCL12 supply from the niche BM mesenchymal stromal cells [76].

### 3.2. Bone Marrow Niches for Normal Plasma Cells

Plasma cells require extrinsic factors for their migration to and survival in the BM [5,8,77,78]. As mentioned above, BM mesenchymal stromal cells secrete high levels of CXCL12 [79,80,81], which is a critical factor for attracting and lodging CXCR4^+^ normal plasma cells in the BM [8,9,82]. Thus, plasma cells from chimeric mice reconstituted with CXCR4-deficient fetal liver cells failed to accumulate in the BM [9], and specific deletion of CXCR4 in mature B cells led to a severe reduction in plasma cells in the BM [10]. Notably, using CXCL12-GFP knock-in mice, Tokoyoda et al. demonstrated that plasma cells are in contact with processes extended from CAR cells [10], suggesting that these cells constitute a favorable niche for normal plasma cells. Whether other stromal cell types also expressing CXCL12 such as LPR^+^ cells, *Nes*-Cre^ER^ or *NG2*-Cre^ER^ cells represent lodging niches for plasma cells remains to be investigated.

Adhesion receptors provide plasma cell attachment to the BM microvasculature, as well as retention at appropriate niches. The Shp phosphatase activates the α4β1 integrin on plasma cells, stimulating their homing to the BM via cell attachment to VCAM-1 [83]. Blockade of the integrins α4β1 and αLβ2 (also called LFA-1) leads to a depletion of long-lived plasma cells from their BM niches [84,85]. The BM stromal cells that mediate the lodging of plasma cells via α4β1 are positive for the expression of VCAM-1 [10,86]. Fibronectin harbors a α4β1 binding motif called CS-1 [87], and it has also been shown to provide plasma cell attachment and survival in BM niches [88]. The αLβ2 ligands on the stromal cells which could facilitate plasma cell lodging are currently unknown.

Several soluble mediators are needed for plasma cell survival and proliferation at suitable BM niches, including IL-6, as well as the B-cell maturation antigen (BCMA) ligands: a proliferation-inducing ligand (APRIL) and B-cell activating factor (BAFF) [5,8,78]. Different cell types supply these factors to the plasma cells. Recently, IL-6 was found to be mainly produced by perivascular cells in the BM [89], but other mesenchymal stromal cells might also contribute to IL-6 expression, especially upon contact with plasma cells [84,90]. Both IL-6 and APRIL are also produced by eosinophils [91], which was suggested to be required for the maintenance of plasma cells in the BM niche. Yet, two other reports concluded that eosinophils were not needed for plasma cell survival in the BM [92,93]. Megakaryocytes are also a source of IL-6 and APRIL in the BM, and notably, megakaryocyte-deficient c-mpl mice display a reduction in plasma cell numbers [94], indicating that megakaryocytes are critical components of the plasma cell BM niche. The involvement of the BAFF–BCMA-dependent signaling on plasma cell survival has been suggested [95], but it remains to be formally demonstrated.

### 3.3. Bone Marrow Niches for Myeloma Cells

#### 3.3.1. The Premetastatic Niche

The BM microenvironment not only regulates HSC and HPC homeostasis but can also promote the expansion of hematologic tumor cells [6,7]. The BM stromal cells and ECM components are essential for the establishment of premetastatic niches, which attract and maintain incoming tumor cells. Upon lodging, the malignant cells locally transform the BM niche phenotype through altered release of cytokines and growth factors, and by remodeling adhesion receptor-mediated cell–cell contacts, ultimately educating their own growth and survival at the expense of the rest of the cell community of the microenvironment [6,41].

The presence of genetic mutations in BM stromal cells has been proposed to generate a local BM region with selective growth pressure in favor of tumor cells, as was demonstrated during BM transplantation [41]. As an example, transplant studies in RARγ^−/−^ mice displaying myeloproliferative syndromes revealed that this disease was non-hematopoietic cell-intrinsic instead of being hematopoietic cell-autonomous [96], which involved a TNF-α-triggered pro-inflammatory environment, and therefore suggesting the participation of the BM microenvironment in hematopoietic disorders. Another example is represented by activating mutations in *PTPN11* in BM MSCs, which promote the development and progression of myelomonocytic leukemia [97]. In this case, secretion by BM MSCs of the chemokine CCL3 stimulated the recruitment of inflammatory monocytes, causing an increase in inflammation based on IL-1β activity, which favored the expansion of BM MSCs, osteoblasts, and fibroblasts [97]. Together, these data reveal that the induction by BM stromal cells of an inflammatory microenvironment contributes to malignant cell growth [6,7]. Whether premetastatic niches harboring MSCs with genetic alterations previous to MM cell lodging could predispose to the survival and expansion of MM cells remains an interesting possibility to be addressed.

Exosomes are intraluminal vesicles of the multivesicular bodies, which are formed by invagination and budding of the late endosomal membrane. They are released after the fusion of multivesicular bodies with the plasma membrane and differ from other extracellular vesicles by their small size (30–150 nm) [98,99]. Exosomes represent a source of local and long-distance transfer of molecular information that can reach cell components of the BM microenvironment, and which might alter their phenotype to foster a suitable premetastatic niche for expansion and drug resistance of arriving tumor cells [98,99]. These vesicles are released by all types of cells in the body, including MSCs, stromal, and endothelial cells, fibroblasts, osteoclasts, osteoblasts and immune cells [98,100]. Exosome cargo includes DNA, mRNAs and miRNAs, integrins, growth factors, signal transduction molecules, and metabolic enzymes [100]. Vascular disruption and leakiness, angiogenesis, suppression of immune responses, and alterations in the composition of the ECM represent common responses promoted by exosomes in the premetastatic niche [98]. In MM, it has been shown that MM cells alter BM-derived cells to secrete exosomes that generate a welcome and growth-supporting environment that stimulate the dissemination of the malignant plasma cells [101,102]. Furthermore, exosomes influence the migration of pre-osteoclasts as well as osteoclast differentiation, involving activation of CXCR4-dependent signaling that leads to upregulation of osteoclast markers [103]. As pointed out above, exosomes carry and deliver miRNAs to target cells. Roccaro et al. showed that the miRNA content in exosomes from MM-MSCs was different from that of normal MSCs, with a higher content of oncogenic proteins, cytokines, and adhesion molecules [101]. Moreover, they showed a reduction in miR-15a in MM-MSC exosomes compared to normal counterparts. In addition, it has been reported that exosomal miR-135b shed from hypoxic MM cells stimulates angiogenesis by targeting factor-inhibiting HIF-1 [104].

#### 3.3.2. Finding the Right Niches

After the adhesive and migratory events controlling MM cell entrance into the BM microenvironment and trafficking inside the BM [16], malignant cells must find suitable niches, including premetastatic ones, for their survival, proliferation, and resistance to chemotherapy. Experimental evidence suggests that tumor cells compete with normal hematopoietic cells for niche occupancy [105,106,107], though they seem to become independent from niche control during disease progression. MM cells probably use identical cellular and extracellular components in the BM microenvironment as their normal plasma cell counterparts to look for and develop a favorable niche. A CXCL12-rich environment is a likely niche for attraction and retention of CXCR4^+^ MM cells. Notably, blockade of the CXCL12–CXCR4 interaction causes MM cell release to circulation [13,14]. Therefore, CXCL12-expressing mesenchymal stromal cells including CAR cells should be considered constituents of MM cell niches. In addition, similar to normal plasma cells, MM cells become anchored to BM niches where ligands for the α4β1 and α5β1 integrins, as well for CD44, are expressed [31,32].

Patients with MM have a pathological imbalance with depletion of osteoblasts in favor of proliferation and activation of bone-resorbing osteoclasts [31,108,109,110]. Like solid tumors displaying BM tropism [111], it has been proposed that the BM osteoblastic niche might facilitate the dormancy of MM cells, whereas the vascular niche could promote tumor cell proliferation in the BM [112]. Thus, it was recently shown that MM cells colonize and reside in the bone niche, in close proximity with osteoblasts [112]. This osteoblastic niche stimulates the survival of MM cells by triggering a dormant or quiescence state, which is turned off upon remodeling of the endosteal niche by osteoclasts, leading to reactivation of MM cells and their displacement from the niche [113,114]. Therefore, extrinsic factors remodeling the niche contribute to the release of these cells from dormancy and MM cell expansion.

Likely contributions to the suppression of osteoblastic activity in BM niches and to the induction of lytic bone lesions in MM patients include the deregulation of the Wnt and Notch signaling pathways, and the blockade of Runx2-dependent functions [115]. For instance, DKK1, an MSC-secreted Wnt pathway inhibitor and whose expression is elevated in BM from MM patients, binds to the LRP5/6 receptor, thus preventing Wnt signaling, and leading to β-catenin translocation to the nucleus and ultimately causing bone lesions [116,117,118]. Furthermore, Wnt5, an abundant growth factor in the BM of MM patients, binds to its receptor ROR2 and mediates MM cell interactions with BM stromal cells [119], and notably, ROR2 depletion leads to detachment of MM cells from their BM niche [120]. Interestingly, inhibition of Notch signaling leads to a reduction in CXCR4-dependent MM cell homing to and infiltration in the BM [121], and Dll1- and Jagged1-induced Notch activation accelerates MM disease by promoting MM cell proliferation [122,123]. With regard to Runx2, its osteoblastogenic activity is suppressed by MM cells, causing osteolysis [109,124]. This suppression is in part mediated by MM cell–osteoblast contacts dependent on α4β1–VCAM-1 interaction [29,124], and also by IL-7 [124].

#### 3.3.3. Educating the BM Niche

Upon stabilization of the MM niche, malignant plasma cells reprogram the local BM microenvironment, either by direct contact with stromal, endothelial or osteolineage cells, or involving the stimulation of supportive cytokines for MM cells, and by facilitating immune evasion [6,7]. These responses provide further expansion signals for MM cells, which become gradually independent from initial normal niche support, leading to the generation of a favorable TME. Notably, MM-MSCs display distinct gene profiles from normal MSC counterparts [125,126]. Furthermore, as mentioned above, MM cells can now act at a distance to prepare further premetastatic niches for tumor dissemination in other bone regions, by releasing cytokines, growth factors, and exosomes that remodel the ECM at these new sites [32,98,101,127].

The direct contacts between MM cells and the surrounding stroma stimulate several signaling pathways such as PI3K/Akt, MAPK, Wnt and Notch, and the induction of NF-κB signaling, leading to upregulation of IL-6, VEGF, IGF-1 and GDF15 expression, as well as increased expression of anti-apoptotic proteins [25,31,32,108,128,129]. In addition to the non-hematopoietic cell components, the TME encompasses hematopoietic cells which might have anti-tumor functions such as T cells and NK cells, or tumor-promoting activity including macrophages, myeloid-derived suppressor cells (MDSCs) and regulatory T cells (Tregs) [32,127,130,131,132]. The complex relationships between MM cells and the cellular components, exosomes, ECM and soluble factors of the TME, overall generate a permissive microenvironment favoring MM cell growth and disease progression. Excellent reviews specifically describing the role of non-hematopoietic cells in MM development and progression have been recently presented [32,127,133,134], and thus we are not discussing it in the present review.

## 4. Myeloid Cells Involved in MM Progression

### 4.1. Macrophages

**Figure 2:**
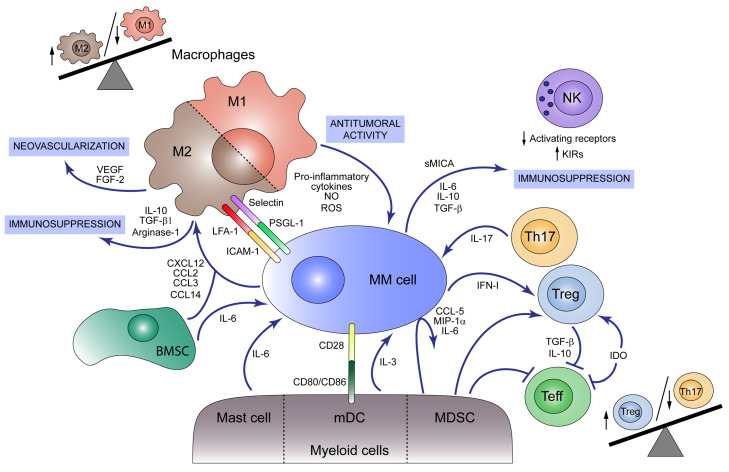
Immunosuppressive tumor microenvironment (TME) in multiple myeloma (MM). Both myeloid and lymphoid cells present in bone marrow (BM) participate in MM development. M1 macrophages act as antitumoral agents producing pro-inflammatory cytokines, nitric oxide (NO) and reactive oxygen species (ROS) and acting as antigen-presenting cells (APC) that activate immune response against MM cells. MM cells and BM mesenchymal stromal cells (BMSCs) produce the chemokines CXCL12, CCL2, CCL3, and CCL14, which promote macrophage M2 polarization and migration to the tumor niche, unbalancing M1/M2 ratio towards the M2 population. M2 macrophages play a pro-tumoral role secreting immunosuppressive agents such as IL-10, TGF-β1 and Arginase-1 and neovascularization factors like VEGF and FGF-2. M2 macrophages act as MM cell protectors through the expression of selectins and LFA-1 in the macrophage surface, which binds to PSGL-1 and ICAM-1 in the plasma cell, respectively. Mast cells, although contributing to an initial tumoricidal host response, secrete IL-6 which promotes MM cell growth. Myeloid dendritic cells (mDCs) foster MM cell proliferation and survival by cell–cell interactions with tumor cells via CD80/CD86-CD28 and secretion of IL-3. Additionally, myeloid-derived suppressor cells (MDSCs) induce the secretion of cytokines such as CCL5, MIP-1α and IL-6 by MM cells and modulate the cytotoxic T cell responses inhibiting effector T cell functions and activating regulatory T cells (Treg). Other signals that promote Treg cell expansion are IFN-I, produced by MM cells, and indoleamine 2, 3-dioxygenase (IDO) activity which at the same time inhibits effector T cells. Cytokines produced by Tregs, such as IL-10 and TGF-β, together with other MM cell-derived signals attenuate effector T cell function. Th17 lymphocytes are also present in BM from MM patients and foster tumor growth through IL-17 secretion. NK cell cytotoxic activity is modulated during disease progression by cytokines and soluble factors (e.g., sMICA) secreted by MM cells that induce a reduction in activating receptors and an increase in inhibitory receptors (KIRs).

Macrophages are one of the main components of the tumor microenvironment in MM [135]. They are terminally differentiated myeloid cells derived from monocytic precursors and can be typically divided into two subgroups according to their functional role. The “classically activated” or M1 macrophages act as antitumoral agents by secreting pro-inflammatory cytokines, reactive oxygen species and nitric oxide while expressing high levels of MHC Class II. On the contrary, “alternatively activated” or M2 macrophages, in which tumor-associated macrophages (TAMs) are included, play an immunosuppressive role that facilitates tumor progression and are characterized by high expression levels of scavenging (CD163) and mannose (CD206) receptors, arginase and the production of IL-10, VEGF and matrix metalloproteinases (MMP). The M1/M2 polarization depends on the activation signaling present in the environment: Th1-derived cytokines such as interferon-γ (IFN-γ) and bacterial products, including bacterial lipopolysaccharides (LPS), promote M1 differentiation, whereas Th2-derived cytokines like IL-10 and glucocorticoid hormones drive the differentiation of macrophages towards an M2 phenotype [136,137]. In MM, it has been reported that both tumor cells and BM mesenchymal stromal cells produce chemokines such as CXCL12, CCL2, CCL3, and CCL14 that promote macrophage migration to the tumor niche and polarize macrophages towards an M2-like phenotype in vitro (Figure 2) [138,139,140]. Moreover, inhibition of the Jak1/2 pathway by Ruxolitinib or blockade of the colony-stimulating factor 1 receptor (CSF1R) seems to inhibit the differentiation of these pro-tumoral macrophages [139,141,142].

Several clinical studies associate high infiltration of M2 macrophages in BM from MM patients with poor prognosis and worse response to chemotherapy and autologous stem cell transplantation (ASCT), whereas patients with high infiltration of M1 macrophages showed better outcomes [139,143,144]. In fact, although M2 macrophages can be detected in BM from MM independently of the stage disease, the grade of infiltration and its correlation with poor prognosis is notable within relapsed MM patients compared to patients with MGUS or SMM [138,145]. In line with these data, some serum receptors such as soluble CD206 and CD163, as well as chemokines like CCL2 and MIF have been proposed as biomarkers for disease progression, prognosis and treatment response [38,146,147,148].

M2 macrophages play key roles in myeloma progression, and they are involved in the emergence of drug-resistant tumor cells. In vitro studies have shown that TAMs induce myeloma cell proliferation and protect them from chemotherapy-induced apoptosis [135,138,141,149,150]. Despite the positive outcomes obtained in MM patients with bortezomib treatment, results showed that this proteasome inhibitor also promotes the accumulation of pro-inflammatory macrophages and fosters MM cell survival and aggressiveness linked to MM disease progression, which could represent one of the mechanisms associated with MM resistance to bortezomib [151]. Moreover, depletion of macrophages or blockade of M2-polarization in murine MM models inhibits tumor growth and disease progression in vivo, and enhances survival after stem cell transplantation, providing strong evidence that macrophages are important for myeloma progression and a promising therapeutic target [142,152,153].

Understanding the mechanisms by which macrophages protect myeloma cells and support their survival will provide new targets for anti-MM therapies. The inhibition of the interaction between myeloma cells and macrophages via PSGL-1/selectins and intercellular adhesion molecule-1 (ICAM-1)/LFA-1 compromised macrophage-mediated protection in vitro, suggesting that cell–cell contact between surface proteins in MM cells and macrophages is important for macrophage-mediated protection of myeloma cells from chemotherapy-induced apoptosis [22,135,150]. Macrophages are also involved in neovascularization through vasculogenic mimicry [154]. VEGF and FGF-2 along with other pro-angiogenic factors promote macrophage recruitment and activation to mimic MM endothelial cells and collaborate with them in vessel formation [155,156]. Furthermore, it was shown that macrophages play a direct role in MM tumor cell homing and migration in vitro [153], while they inhibit T cell proliferation by generating an immunosuppressive environment that favors tumor development [138].

Nevertheless, macrophages can play an opposite role as tumoricidal agents. Infiltrating macrophages can act as antigen-presenting cells (APC) exposing myeloma antigens that activate Th1 CD4^+^ lymphocytes [142,157,158], which reciprocally induce macrophage differentiation to an M1-phenotype by an IFN-γ-dependent mechanism. These M1 macrophages induce myeloma tumor cell death by activation of the intrinsic apoptotic pathway [159], and secrete CXCL9 and CXCL10, two angiostatic chemokines that contribute to the control of tumor development [160]. However, some myeloma cells are able to modulate antigen secretion, leading to the escape from this immunosurveillance mechanism [161,162]. In this sense, macrophage reprogramming by granulocyte macrophage colony-stimulating factor (GM-CSF), added to the blockade of MIF could have a potential anti-myeloma therapeutic effect [163].

### 4.2. Myeloid-Derived Suppressor Cells

Apart from macrophages, some myeloid lineages present in the BM microenvironment play a key role in MM progression. Myeloid-derived suppressor cells (MDSCs), a heterogeneous group of immature cells and their precursors, which can be divided into granulocytic (G-MDSC) and monocytic (M-MDSC), are well-described in the niche of this tumor. Several groups have reported a significant increase in MDSCs in BM from MM patients compared to those from MGUS and healthy donors (HD) [164,165,166,167,168,169]. Likewise, the frequency of M-MDSCs correlates with the amount of M protein detected in serum, and a failure to achieve, at least, a very good partial response (VGPR) after treatment with lenalidomide [164]. By contrast, the use of this common drug in MM has other contradictory actions regarding MDSCs, such as the notable repression of MDSC induction from peripheral blood cells (PBMCs) [170], and the downregulation of programmed death-ligand1 (PD-L1) expression on this myeloid subtype [171]. MDSCs mightily take part in MM progression, as they can induce Treg differentiation [169,170], decrease in T cell proliferation [172], promote MM proliferation [173] and angiogenesis, and even differentiate themselves into functional osteoclasts [165,170]. All these actions could be caused by the MDSCs’ ability to take up MM exosomes through membrane fusion. These vesicles promote MDSC survival by activating STAT-3 and STAT-1 pathways, and by increasing the levels of anti-apoptotic proteins Bcl-xL and Mcl-1 [174,175]. Furthermore, some studies have revealed that MM cells secrete cytokines such as CCL5, MIP-1α [170] and large amounts of IL-6 when MDSCs are present [167,170,173], suggesting that not only do MDSCs contribute to MM progression by direct cell–cell contact or exosome intercellular communication, but also through cytokine secretion.

### 4.3. Other Myeloid Cells

Other myeloid cells such as dendritic cells (DCs), neutrophils, eosinophils, mast cells and platelets are involved in MM development in different ways.

Some studies have demonstrated that both myeloid DCs (mDCs) and plasmacytoid DCs (pDCs) accumulate in BM during the progression from MGUS to MM [176,177], but their functionality is impaired. pDCs from MM BM are incapable of triggering T-cell proliferation compared to pDCs from HD [177,178] and they can promote osteolysis through IL-3 secretion. This cytokine also stimulates pDCs survival and MM cell growth [177]. Migration and secretion of the cytokines IL12p70 and IFN-γ are significantly reduced in vitro in monocyte-derived DCs generated from MM patients. This impaired functionality could be caused by autocrine secretion of IL-6 and activation of the p38 MAPK pathway, both affecting CCR7-dependent migration [179]. Additionally, mDCs support MM proliferation and survival via engagement of CD80/CD86 receptors with their ligand CD28 expressed on MM cells [176].

Neutrophils have also been described to play a role in MM progression. High-density neutrophils (HDNs) present in MM BM exhibit different morphology and phenotype compared to those from HD [180,181]. Five genes (CSK, GSA, MEGF, PGM1, and PROK2), which are especially associated with MGUS-to-MM progression, have been reported to be up-regulated in HDNs from MM patients. Furthermore, these neutrophils show an impaired phagocytosis and oxidative burst in MM [180] while they are able to decrease T cell proliferation, suggesting an immunosuppressive effect of HDNs in the MM microenvironment. Surprisingly, only mature neutrophils, and not any other granulocytic subset, have a significant impact on progression-free survival (PFS) of MM patients [182].

The role of eosinophils in MM progression remains controversial. Although eosinophils have been found in close proximity to plasma cells within the BM [183] and they are known to secrete the soluble factors APRIL and IL-6 [91,92], which somehow could be involved in MM cell proliferation, it remains unclear whether their activity is relevant to BM plasma cells maintenance [91] or not [93,184].

Mast cells and platelets have been also described to have an impact on MM progression. Although mast cells accumulate in the BM to display tumoricidal activity as a host response, they also secrete other factors inducing MM growth, such as IL-6, promoting angiogenesis and participating, mainly indirectly, in MM bone disease [185]. Similarly, platelets are highly activated during MM progression, and their activation status correlates with disease progression from MGUS to SMM and MM [186].

## 5. Lymphoid Cells and Bone Marrow Niche for MM Exacerbation

In addition to the previously discussed subtypes of immune cells, T cell populations are altered in MM patients and switch along with the disease progression. A higher number of Th17 cells has been detected in peripheral blood (PB) and BM from MM patients, and although secreted IL-17 fosters tumor growth [187], an increased population of Th17 cells has been also described in Long-Term Survival (LTS)-MM patients [188]. In this study, Bryant et al. showed that the Treg/Th17 ratio increased in MM patients, but it was lower in LTS-MM subjects. Treg cells produce TGF-β and IL-10, cytokines essential to maintain self-tolerance as they repress effector T cell proliferation. However, an anomalous Treg proliferation or activity could result in immune dysfunction. The role of Tregs in MM has remained controversial and some authors assigned an important but not major role to Treg cells in the progression from MGUS to MM [189]. Nevertheless, analysis of BM-infiltrated T cell populations has detected a higher frequency of activated Tregs in untreated MM patients compared to HD, which was associated with a shorter PFS [190,191,192]. Myeloma-cell-secreted type 1 IFN has been suggested as a mediator of Treg activation and expansion in a syngeneic transplantable murine myeloma model, in which treatment with a blocking IFNAR1 antibody inhibited myeloma progression [193]. Moreover, APRIL, mainly secreted by myeloid cells and osteoclasts, induces proliferation and survival of Tregs in MM [167], and the increased activity found in MM patients of indoleamine 2,3-dioxygenase (IDO), an inducible enzyme that catalyzes tryptophan to kynurenine, led to inhibition of effector T cell function and induction of Treg differentiation [194]. A recently reported subpopulation of Treg CD38^+^ was found to be more immunosuppressive than Tregs CD38^−^ and were decreased in patients treated with daratumumab suggesting an additional mechanism of action for this anti-CD38 antibody used to treat MM patients. [195].

Immune checkpoints have been described as tumor escape mechanisms in different types of cancer. PD-1 and PD-L1 are highly expressed in T cells and plasma cells of MM patients, respectively [196,197]. Furthermore, the expression levels of PD-L1 have been proposed as a prognosis predictor in newly diagnosed MM patients [198]. However, anti-PD-1 blockade therapy has shown limited clinical benefits in MM patients and serious adverse events have been reported in combination with immunomodulatory drugs (IMiDs) therapies [199,200]. The presence of expanded CD8^+^ T cell clones has been associated with good prognosis in MM. Suen et al. described that these lymphocytes express low levels of anergic or exhausted markers (LAG-3, Tim-3, PD-1, and CTLA-4) while displaying a senescent profile (KLRG-1^+^, CD57^+^, CD160^+^, and CD28^−^) [201]. In addition, high-dimensional single-cell RNA-sequencing (scRNA-Seq) and mass cytometry analysis unveiled that T cells in MGUS are enriched in stem-like memory profile whilst MM patients show higher lytic genes (e.g., Granzyme A) and senescence marker expression [202]. Notably, these results contrast with a study from Zelle-Rieser et al. describing both exhaustion and senescence T cell profiles in MM [203]. Additionally, other molecules such as carcinoembryonic antigen-related cell adhesion molecule-6 (CEACAM-6) expressed in MM plasma cells inhibit T cell activation and their cytotoxic response against MM cells [204].

One additional role has been described for T cells in the development of MM lytic bone lesion. Osteoclast differentiation and activation are induced by binding of the receptor activator of nuclear factor-κB (RANK) to its ligand (RANKL) expressed in stromal/osteoblastic cells and activated T lymphocytes. RANKL over-expression in T cells is induced by MM cells [205]. Cytokines such as IL-3 and IL-17 have also been associated with lytic bone lesions in MM [124,206]. In addition, CD4^+^ T cells from MM patients but not from MGUS subjects produce IL-3 [207], a cytokine with both osteoclastogenic and antiosteoblastic effects, and it was shown that IL-17 BM plasma levels correlate with the grade of bone disease [208].

Natural Killer (NK) cells play an important role in immune surveillance against viral infections and cancer. The NK response or tolerance against target cells depends on the balance between stimulatory and suppressive signals through their activating and inhibitory receptors. The reported proportion of NK cells in MM is controversial. Some data revealed a decreased population of NK cells in MM patients [209]. By contrast, other studies showed an increased number of NK cells in BM and PB of MM patient samples [210], although the NK cell cytotoxic activity is reduced and further decreases in advanced stages of this disease [211,212]. Reduction in activating receptors (NKG2D, DNAM-1, CD161) and increased expression of CD158a inhibitory Killer Immunoglobulin-like Receptor (KIR) has been described in NK cells from MM patients [213,214,215,216]. In addition, tumor growth in a murine model of MM induces dysregulation of CXCR3 and CXCR4/ligand expression, which results in a reduction in more functional KLRG1^−^ NK cell subsets in bone marrow [217]. Moreover, NKT cells, an immune population that expresses both T cell and NK cell receptors, also present limited cytotoxic capacity in MM patients [218].

Apart from the aforementioned cytokines and chemokines that regulate MM disease, additional soluble components present in the BM milieu may also be involved in MM exacerbation. Extracellular adenosine (ADO) is an immunosuppressive metabolite whose levels in BM plasma correlate with disease progression in MM [219]. ADO hampers antitumoral immune response through the reduction in CD8^+^ and NK cell cytotoxic function and Th1 CD4^+^ T-cell response, while increasing the proportion of Treg cells [220,221,222,223]. Adenosine triphosphate (ATP), nicotinamide adenine dinucleotide (NAD^+^), and cyclic adenosine monophosphate (cAMP) can be metabolized by ectoenzymatic pathways to generate ADO. The canonical pathway starts from extracellular ATP and involves the ecto-nucleoside triphosphate diphosphohydrolase CD39 and the 5′-nucleotidase CD73 [224]. Furthermore, an alternative route entailing the NAD^+^-glycohydrolase CD38, the ecto-nucleotide pyrophosphatase/phosphodiesterase CD203a and CD73 has also been described [225]. CD39 is localized on the plasma membrane of regulatory T and B cells, and recent studies have also shown its expression in malignant plasma cells from MM patients [226]. Meanwhile, CD38 is expressed in T, B, NK and myeloid cells, and at higher levels in MM cells. Although the ectoenzymes from canonical and non-canonical pathways are present in the MM BM niche, some authors suggested that the hypoxic acidic environment might hinder CD39 enzymatic activity [224], and thus the implication of each pathway for ADO generation in MM has yet to be defined.

Another important factor is the soluble major histocompatibility complex class I-related chain A (MICA), liberated by shedding from the MM cell surface, which downregulates Natural Killer receptor group 2 member (NKG2D) expression in NK and T cells and is a prognostic factor for the overall survival (OS) and PFS of MM patients [227,228].

## 6. Conclusions

The BM microenvironment is essential for MM disease establishment and progression. MM plasma cell trafficking and homing to the BM are regulated by soluble factors, mainly chemokines such as CXCL12, as well as by direct cell–cell interactions through adhesion molecules expressed by plasma and bone marrow-resident cells. Once inside the BM niche, MM plasma cells transform the microenvironment through the secretion of cytokines and growth factors, which along with BM stromal cell activity induce additional mechanisms implicated in MM plasma cell retention, proliferation and drug resistance. Some patients develop extramedullary disease, caused by changes in chemokine receptor expression or function in plasma cells, and also by the MM plasma cell independency of survival factors from BM niche.

The immunosuppressive TME generated by deficient antigen presentation, effector cell dysfunction, proliferation of immunosuppressive cell populations, and high levels of immunosuppressive soluble factors allows the MM cell to escape from the immunosurveillance mechanisms. Targeting the molecular links between MM plasma cells and the bone marrow niche and restoring the homeostasis of the immune system should be crucial steps to cure myeloma disease.
